# Supplementary material for: Putative Biosynthesis of Talarodioxadione & Talarooxime from Talaromyces stipitatus
Source: Molecules. 2022 Jul 13;27(14):4473. doi: 10.3390/molecules27144473 (PMC9318984; doi:10.3390/molecules27144473)
Supplement: Supplementary file 1 [file molecules-27-04473-s001.zip › molecules-1756491-supplementary.pdf]

Putative Biosynthesis of Talarodioxadione & Talarooxime from *Talaromyces stipitatus*

Ahmed J. al Fahad[1]

Supplementary Information

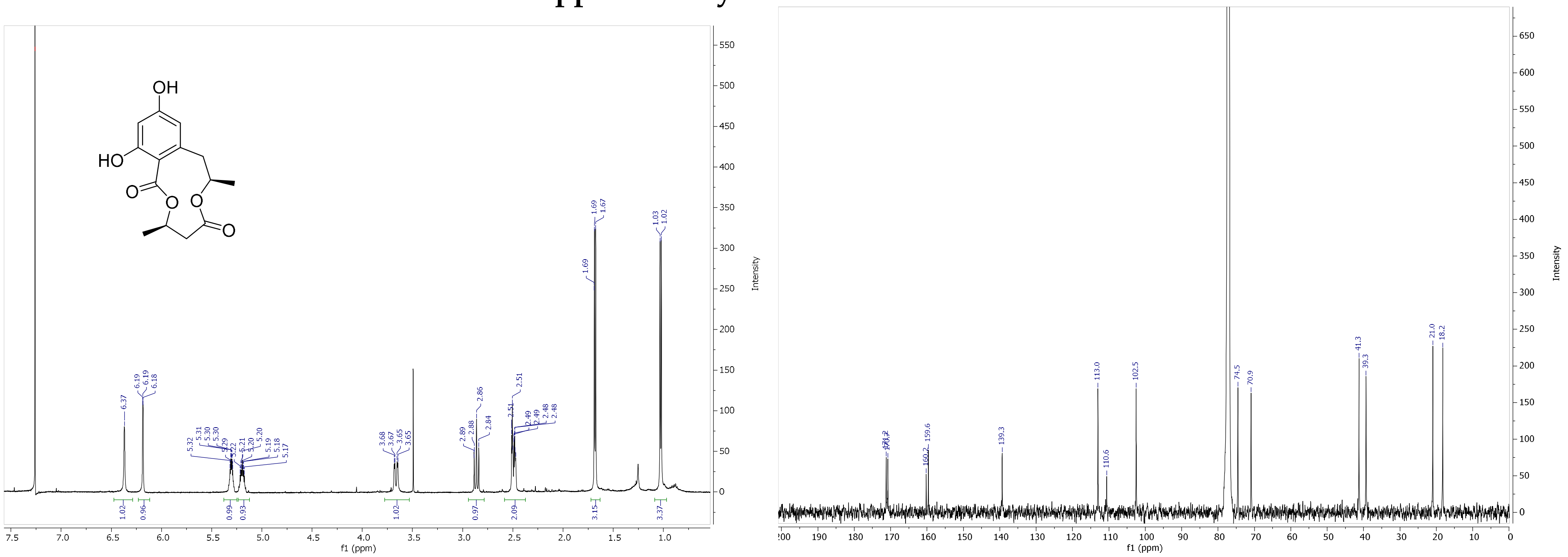

Figure S1. ^{1}H-NMR and ^{13}C-NMR spectra of Talarodioxadione **1** ($CDCl_3$, 600 and 150 MHz).

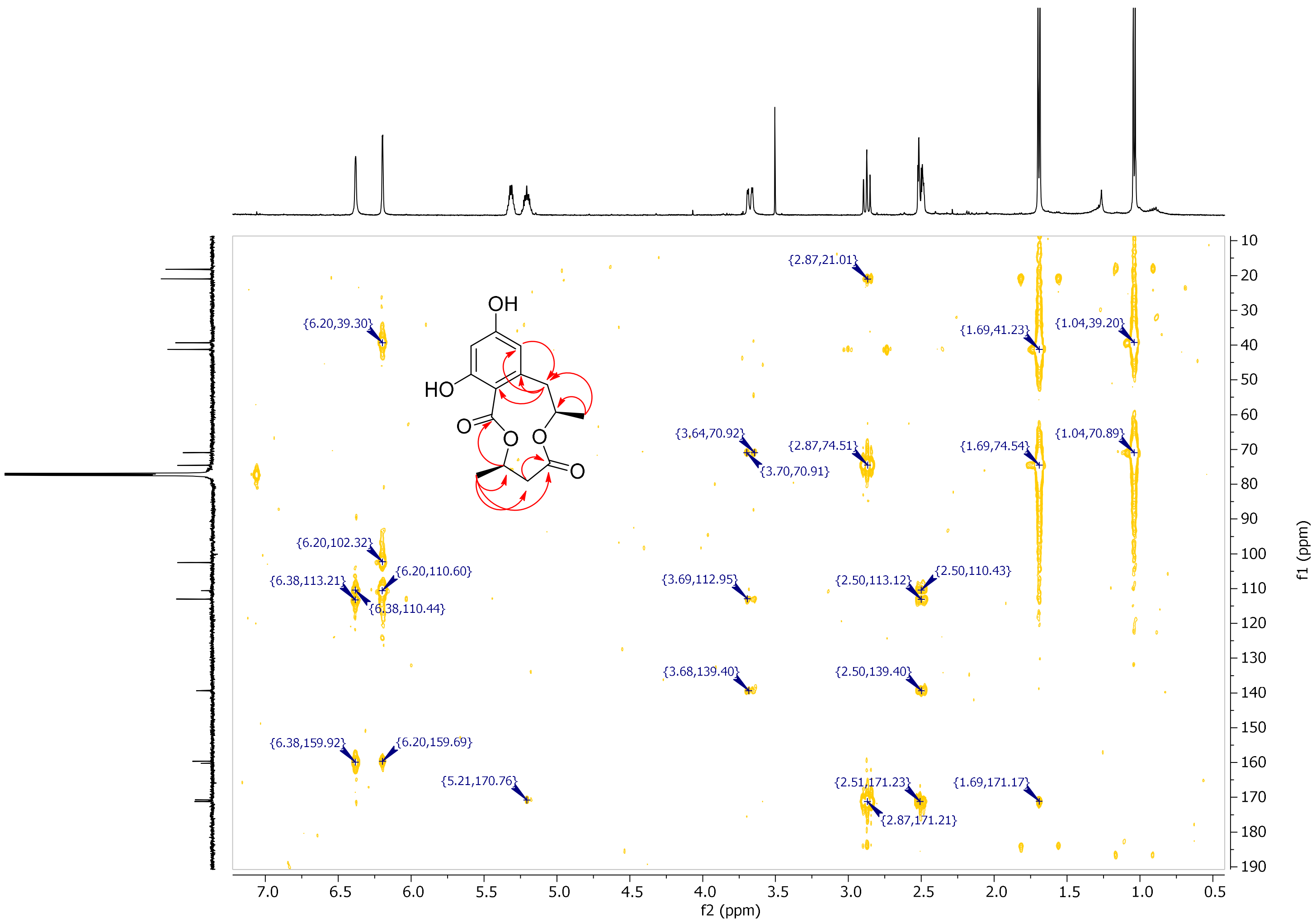

Figure S2. HMBC spectrum of Talarodioxadione **1** ($CDCl_3$, 600 MHz).

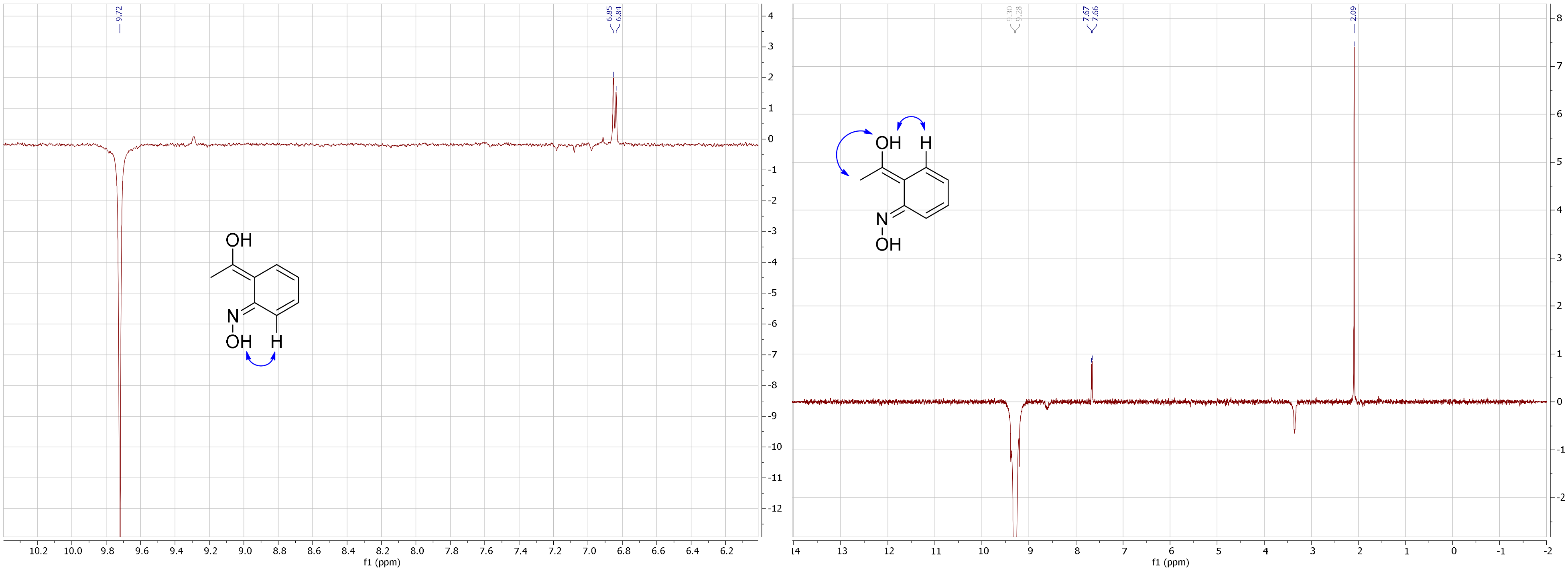

Figure S3. 1D-NOESY spectrum of Talarooxime **5** (DMSO-d_6 , 600 MHz).

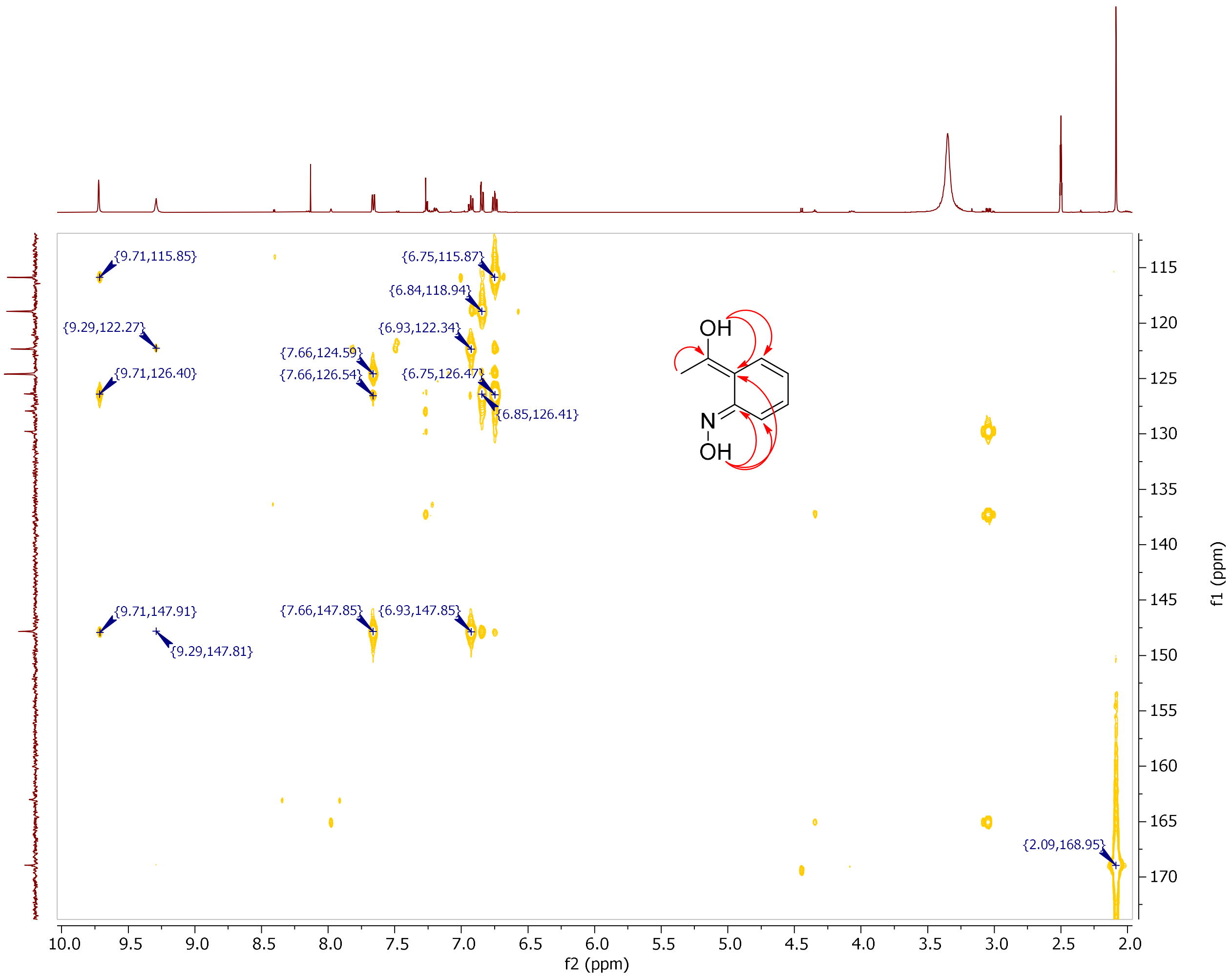

Figure S4. HMBC spectrum of Talarooxime **6** (DMSO-d_6 , 600 MHz).

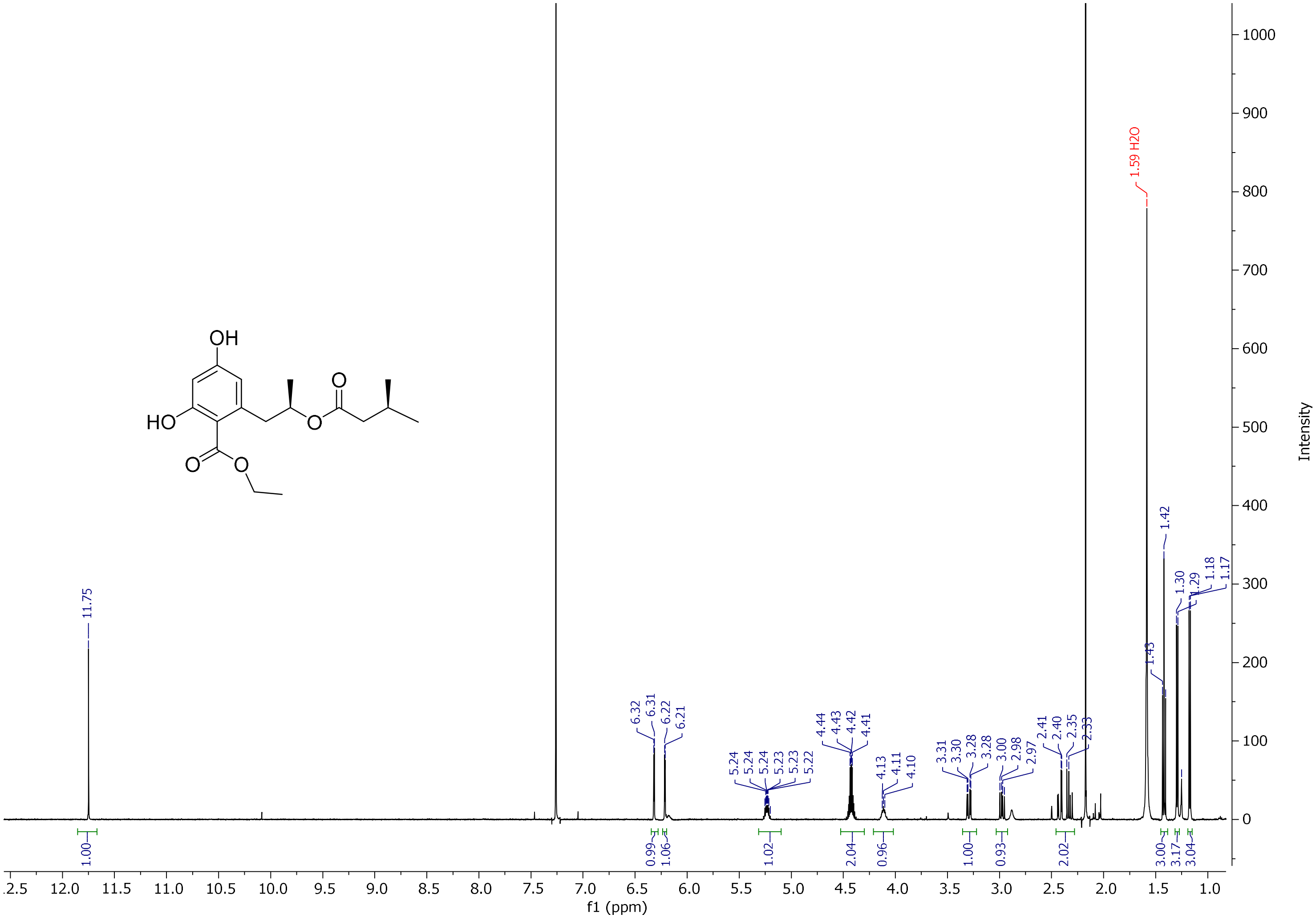

Figure S5. ^{1}H-NMR spectrum of Talapolyester G **2** ($CDCl_3$, 600 MHz).

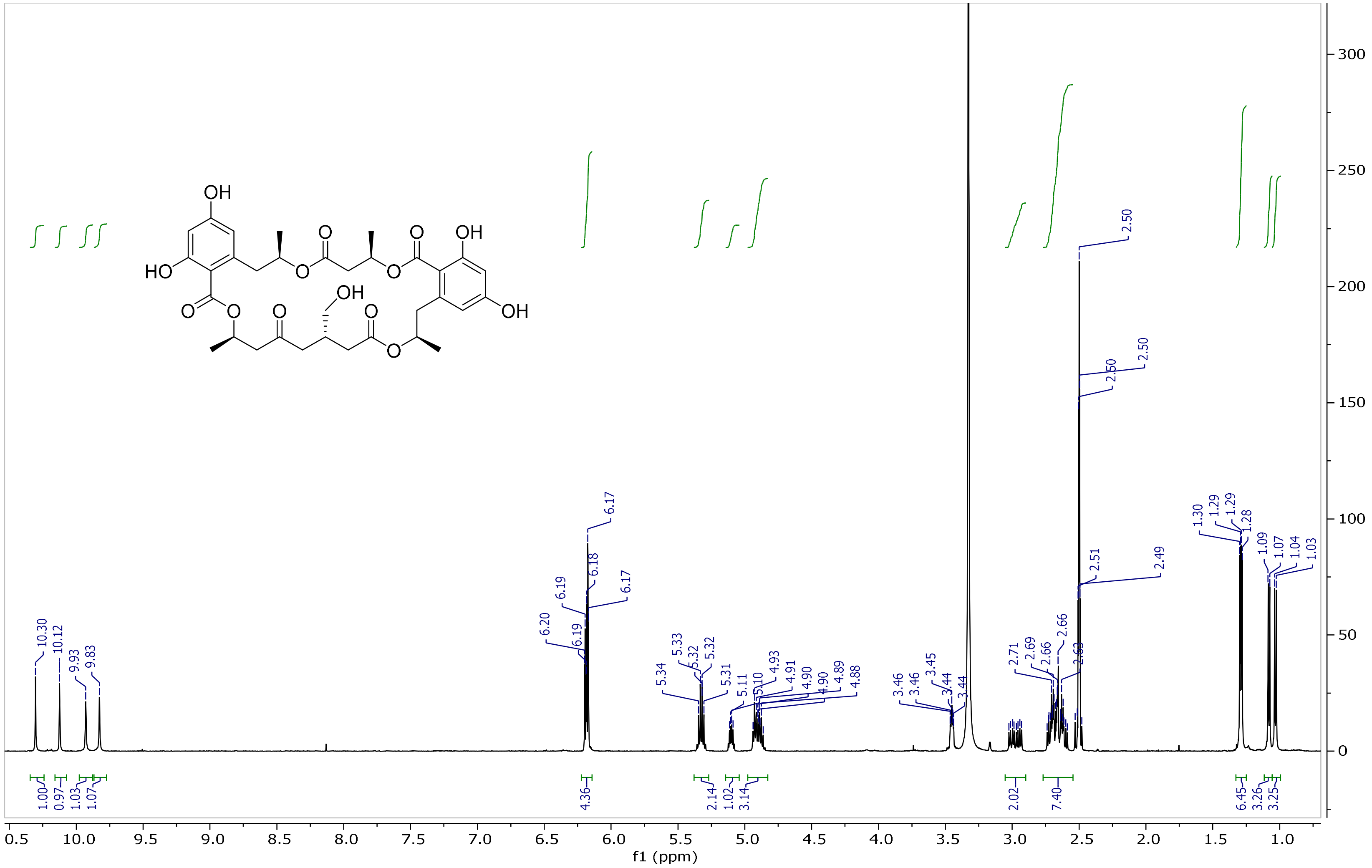

Figure S6. ^{1}H-NMR spectrum of 15G256α **3** (DMSO-d_6, 600 MHz).

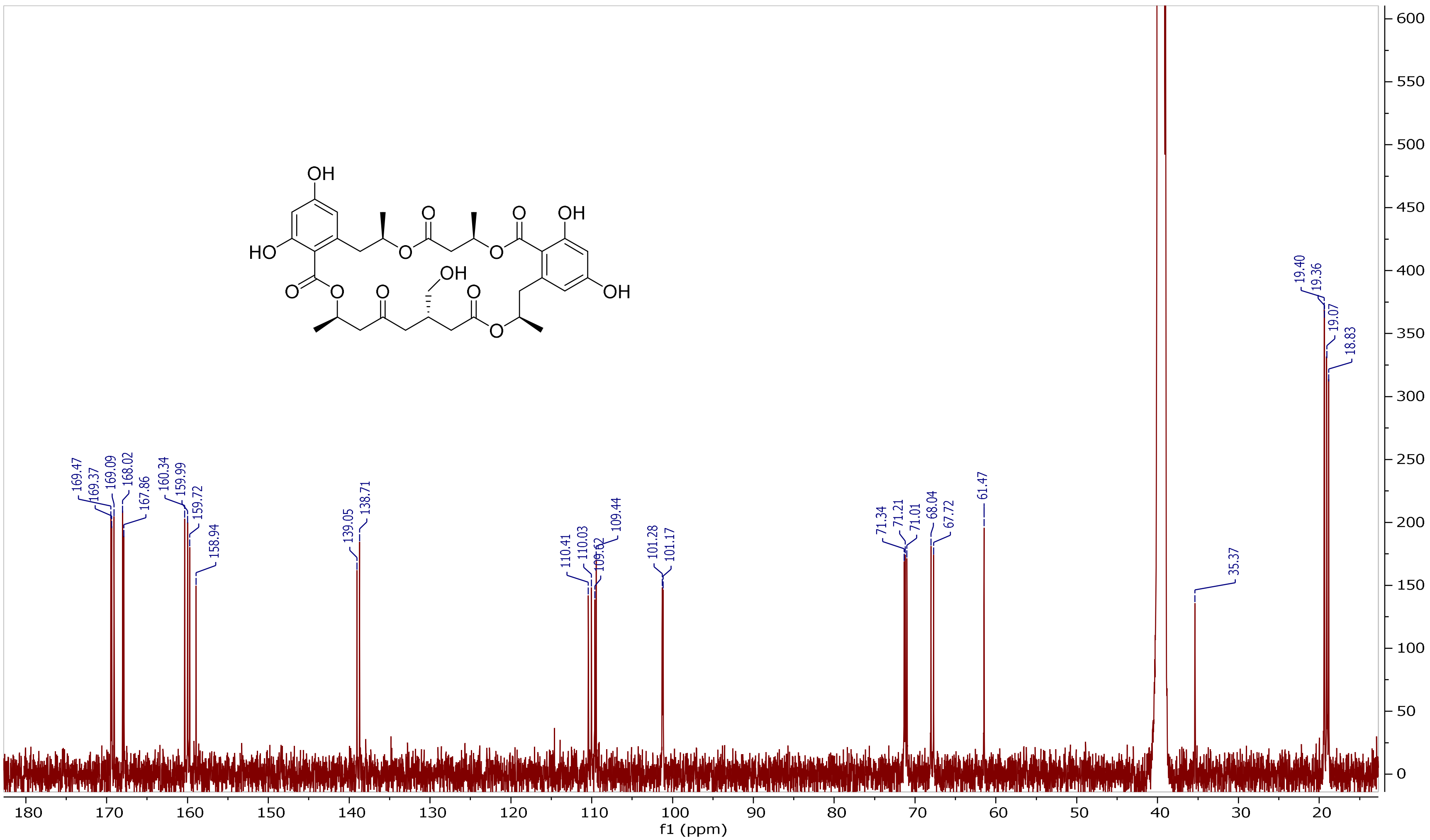

Figure S7. ^{13}C-NMR spectrum of 15G256α **3** (DMSO-$d_{6,}$ 150 MHz).

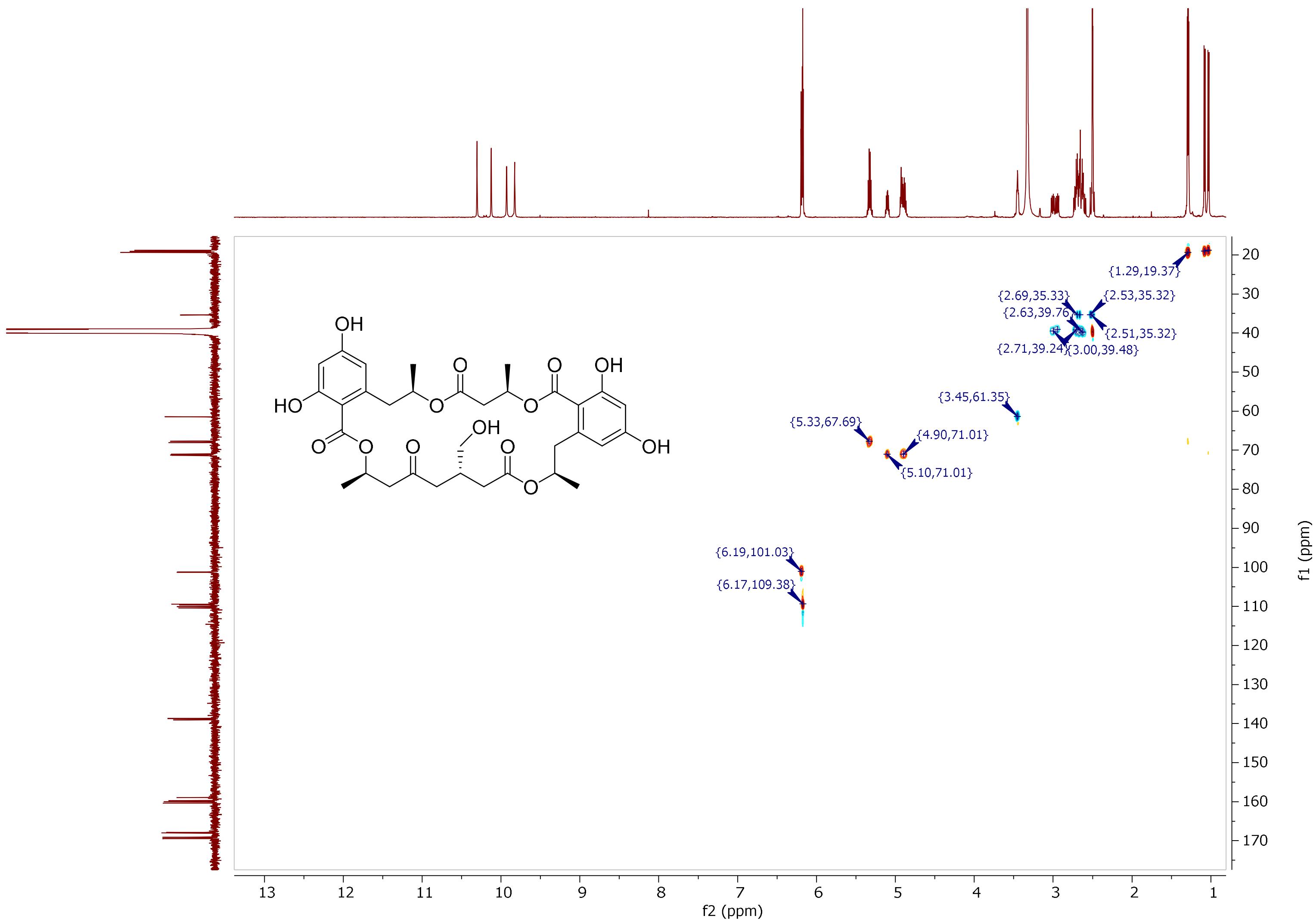

Figure S8. HSQC spectrum of 15G256α **3** (DMSO-$d_{6,}$ 600 MHz).

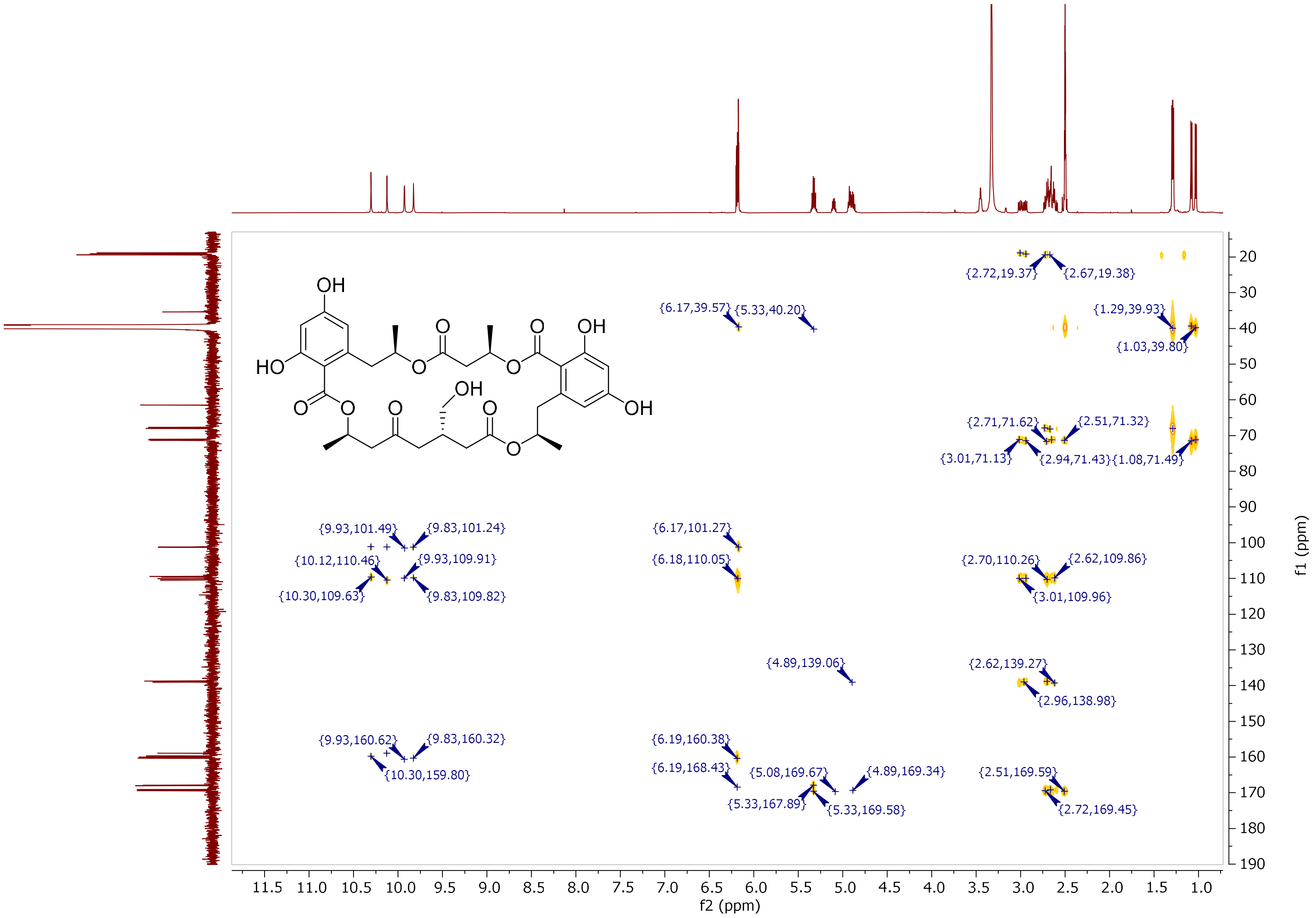

Figure S9. HMBC spectrum of 15G256α **3** (DMSO-d_6, 600 MHz).

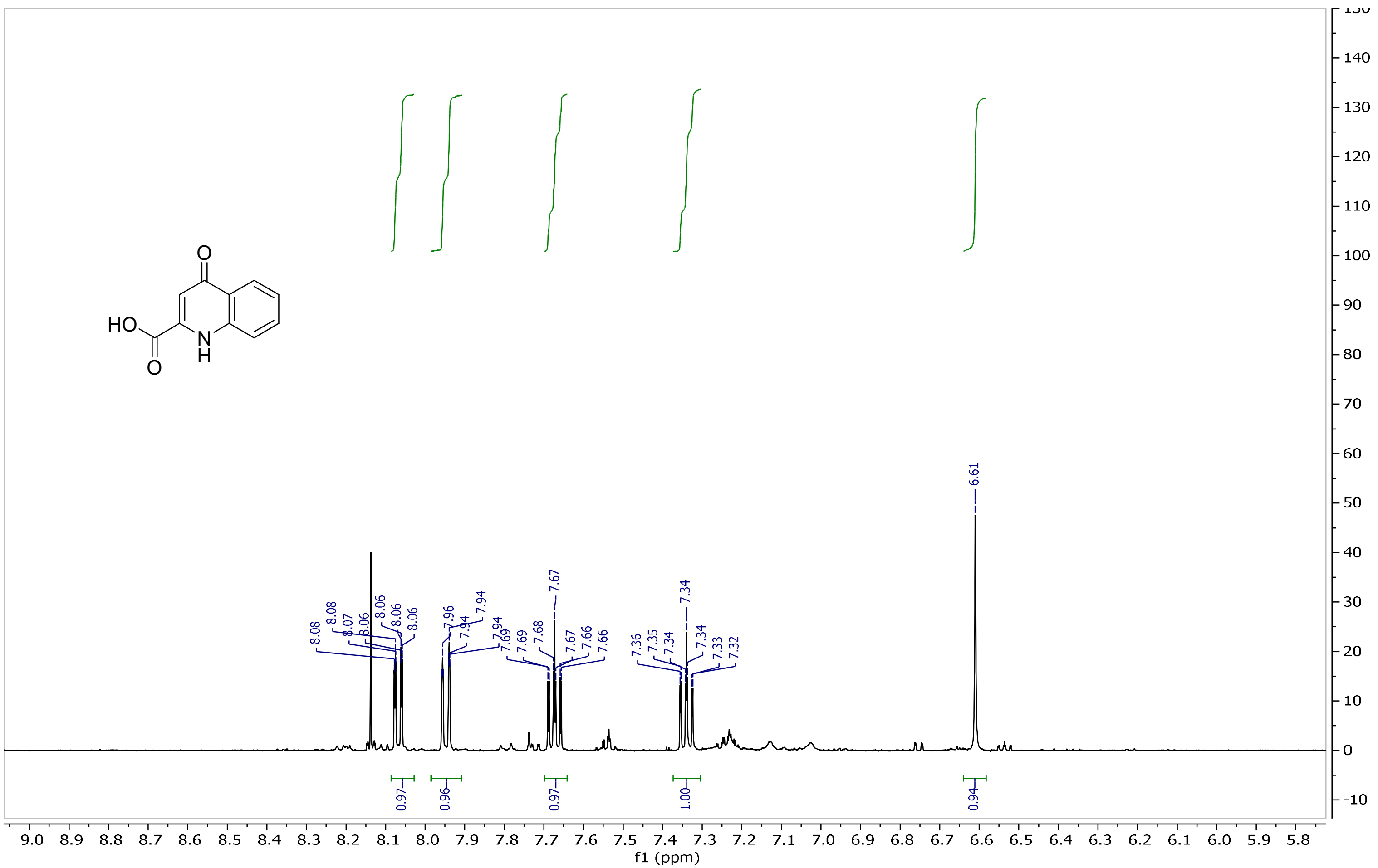

Figure S10. ^{1}H-NMR spectrum of **4** (DMSO-d_6 , 600 MHz).

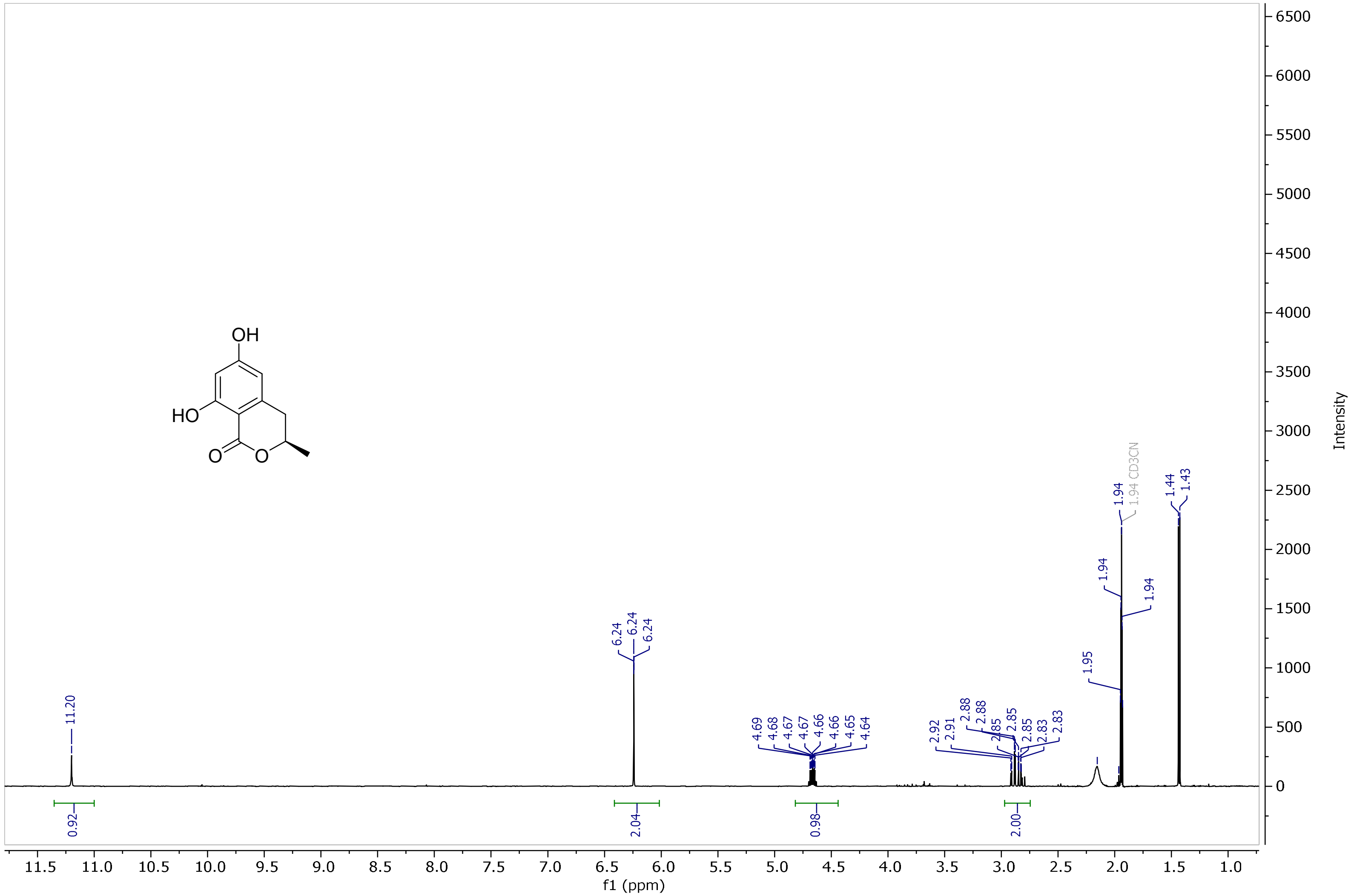

Figure S11. ^{1}H-NMR spectrum of **6** (CD_3CN , 600 MHz).

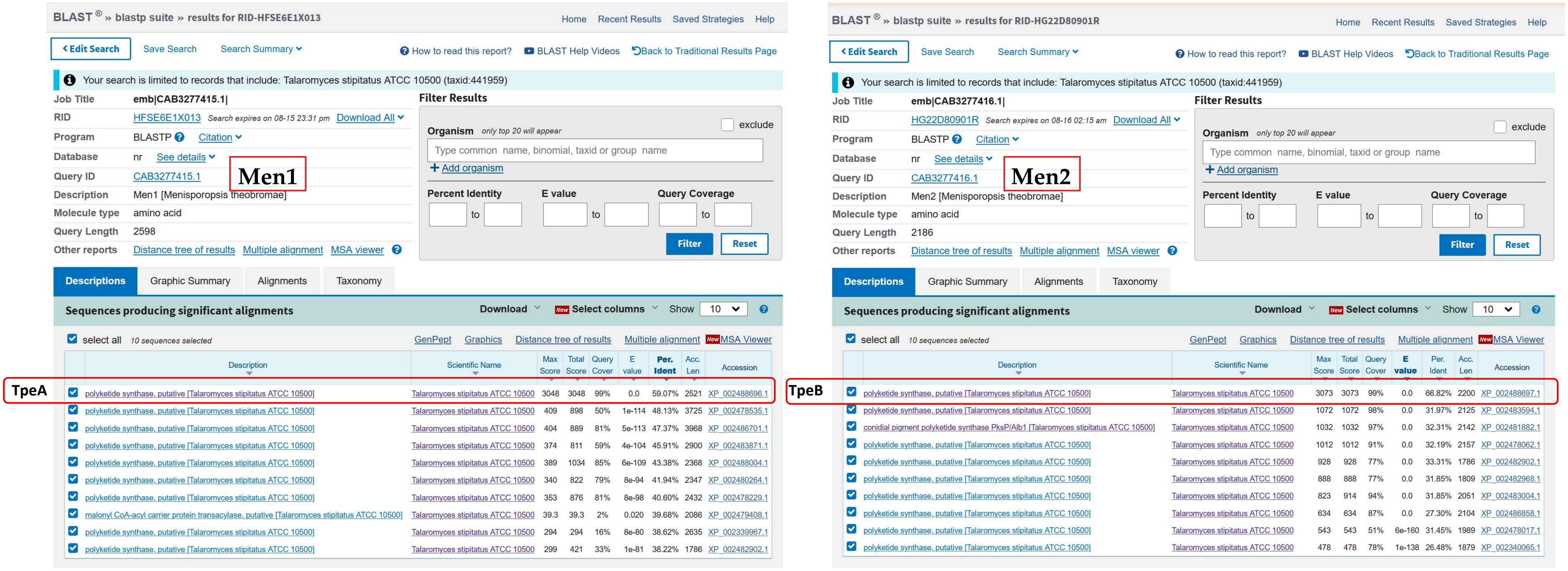

Figure S12. The result of protein Blast searches of Men1 (left) and Men2 (right) amino acid sequences against *Talaromyces stipitatus* protein database.

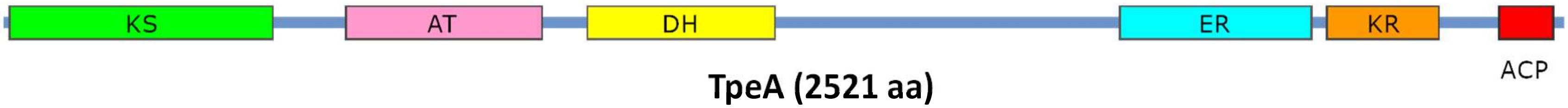

MENSYDDQSIAVIGLSCRFPGDADNVERFWNLLREGQSAISTVPGNRWNSRRFQDDKNHSQNTSRTNRAHFLKEDVSAFDANFFSISKSEAVSMDPQQRLMLEVSYEAFENAGLAVES
LAQSQMGCWVSSFSQDWREMQFSDLQSVPKYAMSGMQPEMLANRVSYFFDLHGPSMALETACSGSLVGLHVACQSLRSGECDAALVGGANLFLNPNMFLALSNQNFLAPDGLSKA
FDASANGYGRGEGFAAVILKPVEKAIRDGDPIRAVIRATGTNQDGRTKGLTMPNGDAQETLIRSTYRSAGLELKDTAYFEAHGTGTQVGDFEELSAIARTVADARKREGLEELWVGSAKTNI
GHLEATAGLAGVLKAILVLENGVIPPNLHFKNPNPRIPFQEYHIRVPTQEVTWNPDTIRRVSVNSFGFGGSNAHAIIDNGKQYLHQRRSKATLVNGTHAADDKELKPEVPQIFVINSSDQEG
LGRQRSALRHYLANFGKEGRANSGWFRDLAFTLGKKRCRLPWRSFCTASTVLELSEALEATDFPKIRSGTAALRLAYVFTGQGAQWAQMGLELFQFPTFKQSVVAADSHLKKLGCPWSV
VEELQRSGAESNIHVSWYSQTLCTVLQVALVELLQSWGITPRSVVGHSSGEMAAAFAIGALAREDAWKIAYWRGKLSSELTERAPDLSGAMMAVGASHEQAQKWVEGLTRGKCVVACI
NSPSSVTVSGDDAGLDELAAMLKEKEVFARKLKVTTAYHSHHMKVVAEAYHDVLKDVEVRNVPTDGPQMFTSVSETLVNPSDLDASHWVANLVSPVLFSNTVAELARAKTPKDQATGS
AVDLMLEIGPHAALRGPVTQILQAKGLRNVEYQSLLSRGCNAIQTTLAAVADLICRGVTADIDAVNNAHTPQGAGHKTMPANTLTTLPPYAWNHSRTFWTESRMLREAKAVNDSPSSFI
GLPMPSFVANEHIWRGFLRLEHVPWVRHHKMQSAVLFPAGGFLAMAIEGASQFQGKDNSRVAKGYKLRDVRIDSAVILTEESNIEHILQLRPHDTARSQQTDSYDGWWEFKISTSAGSD
ESLKCNCYGLVTVEFEQLIHPSHASKASSTTVQQAEAFYERLESVGLEYGPSFQAIKTILHSSGGQTEGEIEIMDIDVNSTTPGGSDGRPYVVHPTTLEALFQMAYAAFDNQSDGVKKALLVT
DIEELLLDASISHTPGARLQTSARSSRLGFREMLADVTVSPTASNAGGISVRGLTCVEMPSTAGVNSDVGITGREGYDSMLSKFVWKPALNLLSATEREHLLIAATKVTDAETEAILAREAAD
FQNVKSALQASQRGKTGSLKLRNALKWISQELPDAKVFGKALENVSQDGQGAGPTGPIVDVLSGTSTPDVLLSGYGSTEKFLAALPGVKVSLQKMYQLVSIMAHENPDLAVLEIGSGSSG
EEDTSIFSTTDIPTTIKYTRAVATPEILRNLQEFSPPAGPIPRFTVLDLEQDLADQGIDLASFDIIIGNNVLSNSRNVENVLRRTKSLLRDEGNMCFVELTKPSSRAVPVLGVVCDWWKRGDDG
LRRPLSSDSIKDILTEQGFTLDFLSPDFVDPAIQQSSLVFASSSAENLPTASATDDADDEEIYILISPELKSPDAVVQNVASSFQHSQIVTWGAGVDFKGKYIISLLELYDPFINHLTEEDFDILKKLV
TQASSLLWVTGIPEPHASTILGFARVVRFEIPSLDFRVLTLDPATIEDAKKNSIHISQVQRSKSPDKEYKELDSIVHIPRVTVDASLNHQISNLCLSEAIESKPLGSLERPQKLCIRNPGMLNTLCF
QADNVSENELQDDEVEVRVMASGLNSKDVMVALGHITDTHLGLEASGVVLRVGASVTHLQLGDKVVMLANGAHRTTLRGKAAVCQKIPQDMTYEEAAGFPFAYCTAYYTLLHVLRAQ
SGQSVLIHAAAGAVGQALVRLAQHLGLEVFTTAESADDRAFVQELLGLFPDRIYHPQDKGLVQHVMRITGDVGVDFIVNPLSVEEIADSLNCLADFGTFVEMGMENDSRHSTLNTRLFRR
DTTFVAVNLQRVLELRPKLIGEILQKALGLFQDGQVKSVSPSNVYPVSEAEAAIQMFQDNRHHGKITLSYSSEDFVSVLQNPKDSLKLSPDNTYLIAGGLGGIGRSLATLLVDCGARHIAMVS
RSGVTSVEQQQLIDNLSERGAKIGVYKCSIGDANALGRALTQCASEMPPIKGIIHSAVVFRDAVLHNMTYKQWDELLDGKLRGSWNLHALTTSYNLDFFLCIGSFMAIIGGISQSNYAAGSA
FQDGLAHLRQSQGLPAVTIDLGIVKGFGAVEEQGAVGHTLEWREPFGVSEPEVHALIKGALLGQRGNWALDVPAQMINSIPTGGMVRSSGVSQPYYFDDPRFSIMAKIGMDSENSDTQ
ASVSLKELLADAESSDDAVLFVTGAVISKVAKLMQVAEEEIDTGKSLHAYGVDSLVAVEYVSWAKKEVSADITVFDVMASKPIMSFARDLVGKGKWGAAIPACKS

Figure S13. The amino acid sequence and domains of TpeA which were identified using SMART as follows: KS (green), AT (pink), DH (yellow), ER (cyan), KR (orange) and ACP (red).

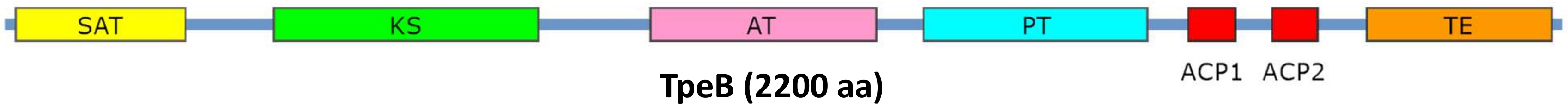

MGSLSSARESAQRITFFGDQTVDTLLCIKDLANRSHQLPILRRYLREAADKLQLLLSQIELGDYECYRNFETIVELAEIYSKQDGTYEPIGCALWTISQFADYLSRSETDPSILTLS
DSAAQPTYVVGVCGGLLLGAAAATARDINELLDIGRKLVDVSFNLGVAQWKRAMDIEGKPGRWAVAIVNVPPKQIRNIITAFNEDMAIPKHRQFYISFLAKGWAAISGPPS
IFPELWEYSSTLSSASKMDLPLGTPAHAAHLRSLNIEELIGSGSVLDLPVRQDRLVISTSTCKPFESQTFGSLLSSSLHDITGETLHIAGVNDYVASCLSRETLVQVSSFGPTSQIG
SFKKALEDSGLKVDLDLSVPESKTPNLLKNPDARDGSNMIAIVGQSVRLPGSDDVKTFWENLKAGQTFESEIPPSRFDLQNYYDTTGAKKSSVTTRFGHFLDRPGLFDCRLF
NVSPREAKQMDPIQRLLLMCSYEALQTAGYSPDSSLSTNRMRVATYFGQSGDDWRQGRASQDVDIYYIPGTIRSFAPGKLNYHYKWGGGNYSVDSACAASTTSVIMAC
NALLGRECDMALAGGGQLHLEPENYAGLSRAGFLSKTTGGCKTFREDADGYCRGEGIGVVVLKRLEDALADNDNVLAVVRGADRNYSWDASSITHPSANAQATVIQRV
LRNTGVEPADIGFVEMHGTGTTAGDSVEMKTVTTVFGSRPKDNPLYIGAVKANFGHGEAAAGITSLLKAVQMLGQKTIPRQPGFPGPKDPTFDHLDAMNIRIPDSSFPFP
TPTRPFSQDGKMRILVNNFDASGGNNCVLLEEAPDKNVSQIQDPRQYYTVAISARTTKSLQKNMERLSDYLVKHPDTPVADVAYTSTARRIHEDLKRSYTVDSTESLVGLLQ
ADLKKDLTGIRPATPRSVVFAFTGQGSQYSGMTKQLFETCTPFRESVQSLHDLAVWQDFPSFLNLFTDDVTDEISASPIQTQLAIVVLEMSLANLWKSWGVEPDLVIGYSLG
EYVALYVSGVLSAHDVLFLVGNRAQLMDERCEIGSYSMLAVQASPEDLEEPLKAYPTSNVACRGAPRSTVVSGPSEDIAKLHAELKEKSISGTILNVPYGFHCAQVDPILEDF
RDLLDRVPFSKPRIPVASSLDGVIVTDDDVFSPSYMVRQTREPVAFVSALKAIETSHLVDNTSLWVEIGPKRVLNSFIKATLAVDHDRLLHSVDEKASNWRTIATAITACWQG
GVSIKWQNFHRQFTKHLRLVDLPTYAFDLKDYWIEPAAPIVQQRSAAEPLRQVAVPAVPGFPTASLQRVREERIQGDNAAVTFESTLSHPDLMGLIRGHQVNGVDLFPAS
GWWDMAYTAAKYIHHRIQPSRGAAPGLSMLDCSITHPLMPSASEDQQKLVIIVAKKQAGSSVVEVSFKSQEKSVEQDHGSCKIRFESKADWEAEWSRSAHFIKAAKNNV
VTNATRPDGNGHKLPKPVVYTLFSNFVNYSGDFKGIQQVYLNADFQREVVADVVLPAGMYNFNLNPYWSDALIHACGFMLHSDPDLPTQDCFLFNGFEELRFLMDDLL
PGVPYTSYVFMHDTNSQEVPAKRTRNVTGDIYIFQGEKIVGTVQGVVFQRLTKRILTTILGKSQDHHNSNEVRNGNATTTHTNPPAHATTQSFFAPPGIKPAVAFSSAPATV
GEETAEAVIAKILTKTGANRASLSESTTLGEIGLDSLEWIELVGVFRSALDIEVPASFFFEYPKVLRLRQAIAELPLEGEKEESGSSSPDSPYGMLTPATGRFTPITASRSTHNSDG
PANYANIVLDIVLSQTGFDKDDVLPSTRFDDMGLDSLCTMEVVSLVREQTGLDLPASFFHHHPTVADVRKNLGPNTEDKSKDSVKASASIAVSEPATELVVVHHPGNSPAP
ESLPIIDEDLKDYHCDFFLMQGSSDSAEIPMFFLPDGTGYPAVLLKLPPVFKGDNPLFTCKSPLLHRAEGREVACTIEGLALSYAEAIRRTRPHGPYLLAGYSLGAAYAYEVAKIL
ADAGEIVQGLLFVDFNMAASVGLEHRERKPVPTNLNVGVMEQVGWMNGIHNDEKNFHIPPAPPKIKFHALSVFKSLTRYFPKPMTPSQRPRNTYALWAGAGMEDLLG
PSNAGFLPEFGIIDWQMGSRRENNGPAGWENFIGGPVRCATVPCDHLSIMMSTDWVGTTANIIKDLLEDALSNPGTP

Figure S14. The amino acid sequence and domains of TpeB which were identified using SMART as follows: SAT (yellow), KS (green), AT (pink), PT (cyan), ACP1 (red), ACP2 (red) and TE (orange).

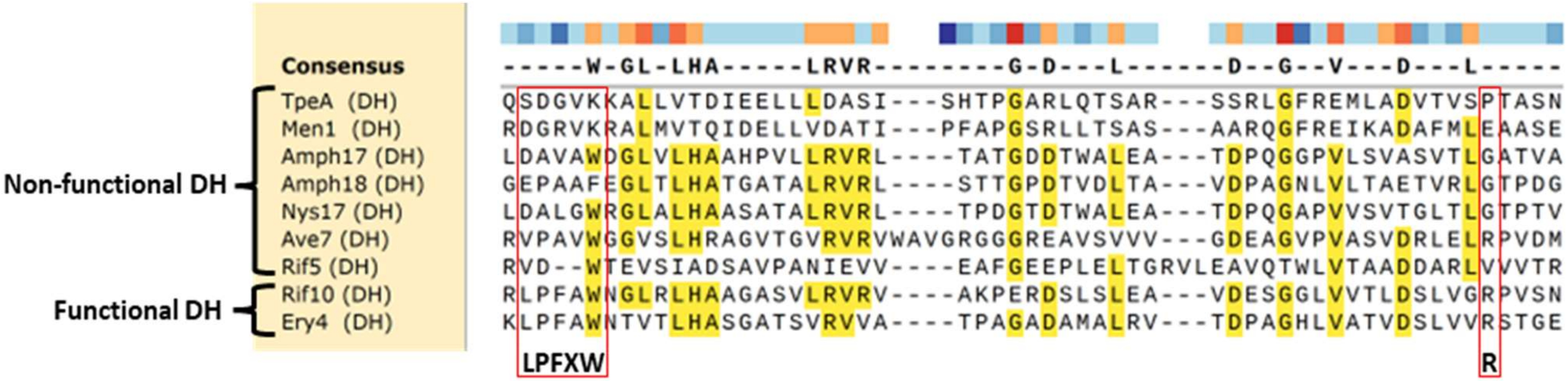

Figure S15 : Protein sequence alignment of the predicted site in DH domains for ACP interaction of TpeA and other functional and nonfunctional DH domains including Men1 and modular HR-PKSs such as Amph17 (AJE44526) and Amph18 (AJE39059) from *Streptomyces nodosus*, Nys17 (AAF71767) from *Streptomyces noursei* ATCC 11455, Ave7 (BAA84478) from *Streptomyces avermitilis*, Rif5 (AAC01711) and Rif10 (AEK39124) from *Amycolatopsis mediterranei* S699 and finaly Ery4 (CAM00064) from *Saccharopolyspora erythraea* NRRL 2338.

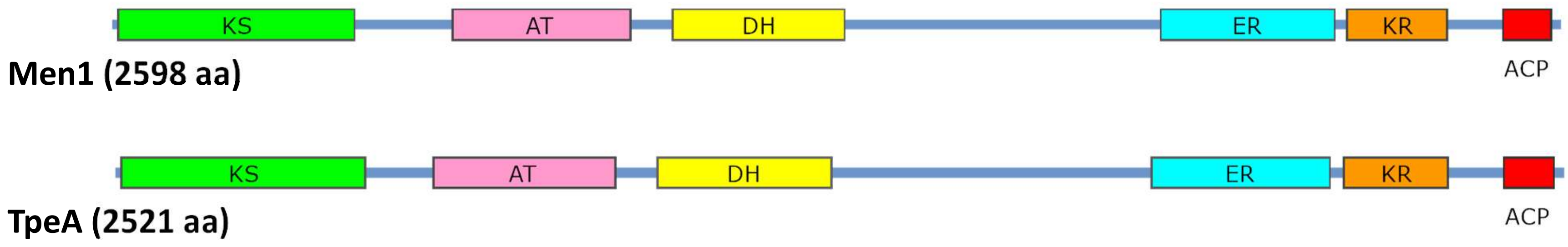

Table S1. The similarity of TpeA domains to their counterparts in Men1.

Domains	Size (aa)	Similarity to Men1 domains	E-value
KS	428	79.72%	0.0
AT	318	76.49%	5e-170
DH	302	50.00%	2e-89
ER	313	42.54%	2e-85
KR	181	77.90%	2e-93
ACP	85	70.59%	7e-32

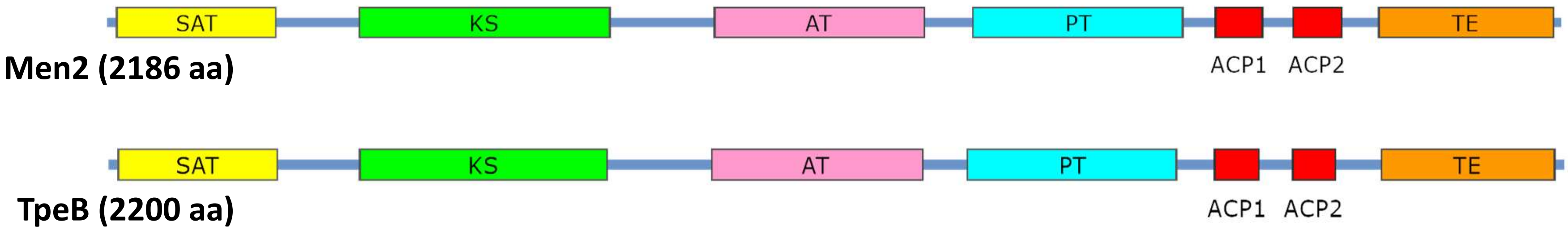

Table S2. The similarity of TpeB domains to their counterparts in Men2.

Domains	Size (aa)	Similarity to Men2 domains	E-value
SAT	241	73.75%	7e-113
KS	376	79.79%	0.0
AT	320	68.75%	4e-159
PT	318	58.62%	5e-134
ACP1	67	64.18%	3e-27
ACP2	65	83.08%	8e-36
TE	262	78.16%	4e-132
